# Multiparametric cardiovascular magnetic resonance surveillance of acute cardiac allograft rejection and characterisation of transplantation-associated myocardial injury: a pilot study

**DOI:** 10.1186/s12968-014-0052-6

**Published:** 2014-07-20

**Authors:** Christopher A Miller, Josephine H Naish, Steven M Shaw, Nizar Yonan, Simon G Williams, David Clark, Paul W Bishop, Mark P Ainslie, Alex Borg, Glyn Coutts, Geoffrey JM Parker, Simon G Ray, Matthias Schmitt

**Affiliations:** 1 North West Heart Centre and The Transplant Centre, University Hospital of South Manchester, Manchester, UK; 2 Centre for Imaging Sciences & Biomedical Imaging Institute, University of Manchester, Manchester, UK; 3 Institute of Cardiovascular Sciences, University of Manchester, Manchester, UK; 4 Alliance Medical Cardiac MRI Unit, Wythenshawe Hospital, Manchester, UK; 5 Department of Pathology, University Hospital of South, Manchester, UK; 6 Christie Medical Physics and Engineering, The Christie Hospital, Manchester, UK

**Keywords:** Cardiovascular magnetic resonance, Heart transplantation, Acute rejection

## Abstract

**Background:**

Serial surveillance endomyocardial biopsies are performed in patients who have recently undergone heart transplantation in order to detect acute cardiac allograft rejection (ACAR) before symptoms occur, however the biopsy process is associated with a number of limitations. This study aimed to prospectively and longitudinally evaluate the performance of multiparametric cardiovascular magnetic resonance (CMR) for detecting and monitoring ACAR in the early phase post-transplant, and characterize graft recovery following transplantation.

**Methods:**

All patients receiving a heart transplant at a single UK centre over a period of 25 months were approached within one month of transplantation. Multiparametric CMR was prospectively performed on the same day as biopsy on four separate occasions (6 weeks, 10 weeks, 15 weeks and 20 weeks post-transplant). CMR included assessment of global and regional ventricular function, myocardial tissue characterization (T1 mapping, T2 mapping, extracellular volume, LGE) and pixel-wise absolute myocardial blood flow quantification. CMR parameters were compared with biopsy findings. As is standard, grade 2R or higher ACAR was considered significant.

**Results:**

88 CMR-matched biopsies were performed in 22 patients. Eight (9%) biopsies in 5 patients demonstrated significant ACAR. Significant ACAR was associated with a reduction in circumferential strain (−12.7 ± 2.5% vs. -13.7 ± 3.6%, p = 0.047) but there was considerable overlap between groups. Whilst trends were observed between ACAR and proposed CMR markers of oedema, particularly after adjusting for primary graft dysfunction, differences were not significant. Significant improvements were seen in markers of graft structure and contractility, oedema and microvascular function over the period studied, although few parameters normalised.

**Conclusions:**

This study provides novel insight into the myocardial injury associated with transplantation, and its recovery, however multiparametric CMR was not able to accurately detect ACAR during the early phase post-transplantation.

## Background

Acute cardiac allograft rejection (ACAR) affects approximately 20% of patients in the first year post-transplantation, and represents a leading cause of death during this period [1]. Moreover, even when apparently successfully treated, an episode of ACAR occurring during the first year confers higher two- and four-year mortality in patients surviving beyond the first year [1].

Clinical features of ACAR are unreliable, with patients usually remaining asymptomatic until hemodynamic complications ensure. Routine screening is therefore performed in order to detect ACAR, and hence augment immunosuppressive therapy, at an earlier stage, with the aim of preventing progression to more severe disease, and potentially reducing the risk of long-term complications. ACAR surveillance is performed via histological analysis of right ventricular myocardial tissue obtained at endomyocardial biopsy, and patients undergo frequent biopsies (10–15) during the first post-operative year. However, the procedure is invasive (complication rate 0.5-1.5%), expensive and disliked by patients, factors which prevent more frequent monitoring, limiting optimal titration of immunosuppressive therapy [2]. Furthermore the sensitivity of the technique is limited by sampling error due to the patchy nature of ACAR and variability in interpretation of the histological appearances [3].

Cardiovascular magnetic resonance (CMR) is a potentially attractive screening modality for ACAR due to its lack of ionizing radiation and its multiparametric nature, i.e. its ability to assess multiple aspects of myocardial injury in a single examination, including global and regional ventricular function, myocardial oedema (quantitative T1 and T2 mapping), myocardial blood flow and focal (late gadolinium enhancement, LGE) and diffuse (extracellular volume; ECV) myocardial interstitial expansion.

Systematic evaluation of multiparametric CMR for diagnosing ACAR has not been reported to date. The aims of this prospective study were to assess the performance of multiparametric CMR for detecting and monitoring ACAR, to characterize the graft injury following transplantation and to longitudinally evaluate graft recovery in the early phase post-transplantation.

## Methods

### Patients and study design

**Figure 1:**
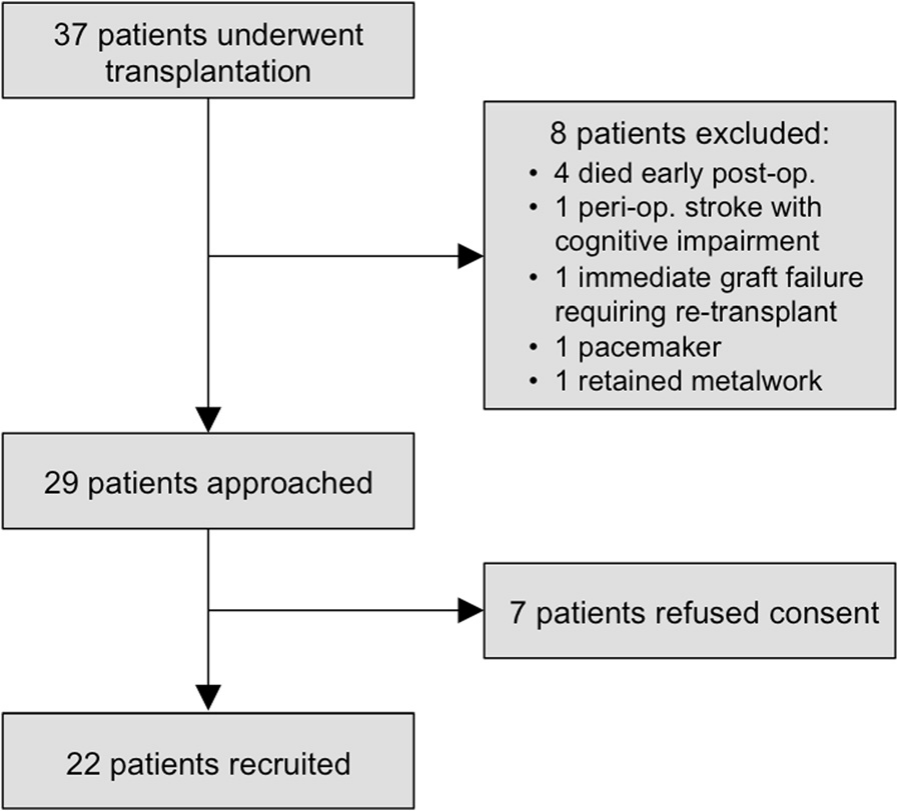
Recruitment data.

All patients receiving a heart transplant at a single center over a 25 month period were prospectively approached within one month of undergoing transplantation (Figure 1). The only exclusion criteria were contraindications to CMR scanning and inability to give informed consent. The work was conducted in accordance with the Declaration of Helsinki, an ethics committee of the UK National Research Ethics Service approved the study (09/H1003/100) and written informed consent was obtained from all participants.

Each patient was prospectively scanned on the same day as biopsy on four separate occasions. As pre-specified, scans were performed to coincide with biopsies scheduled for 6 weeks, 10 weeks, 15 weeks and 20 weeks post-transplant. Investigators were blinded to biopsy results i.e. scans were not performed on the basis of confirmed or suspected ACAR. CMR was performed immediately before or after biopsy, the order of which was determined randomly. Due to concerns over cumulative gadolinium contrast agent dose, the parts of the protocol requiring contrast agent were performed in two out of the four scans in each patient. Patients with an estimated glomerular filtration rate of 35 ml/min/1.73 m^2^ or less did not undergo the parts of the protocol requiring contrast agent. In addition, 10 age- and sex-matched healthy volunteers (asymptomatic, no risk factors, normal physical examination, normal electrocardiogram) were recruited to undergo CMR scanning.

### CMR acquisition

CMR was performed at 1.5T (Avanto; Siemens Medical Imaging, Germany) with a 32-element phased-array coil. Steady-state free precession cine images were acquired in standard long-axis views and in a stack of short-axis slices covering the left ventricle (LV). Short-axis tagged images were acquired at basal, mid and apical ventricular levels using a segmented k-space fast gradient echo sequence with spatial modulation of magnetization in orthogonal planes. A single-shot modified Look Locker inversion recovery (MOLLI) sequence was acquired in short-axis at mid-ventricular level before contrast agent was administered, and 15-minutes after the contrast agent ‘top-up’ (see below) [4]. Same day haematocrit was measured. A T_2_ -prepared SSFP sequence was acquired in short-axis at mid-ventricular level in late diastole at preparation times, T2_prep_, of 0, 24, 45, 65 ms. [5] Perfusion imaging was performed as described previously [6]. Briefly, a saturation recovery gradient echo sequence was acquired during adenosine stress and at rest following a 0.05 mmol/kg bolus of gadolinium-based contrast agent (gadopentetate dimeglumine; Gd-DTPA; Magnevist; Bayer Healthcare, Germany). Following rest perfusion image acquisition, a further 0.1 mmol/kg of contrast agent was administered (‘top-up’) to bring the total dose to 0.2 mmol/kg. Standard late gadolinium enhancement (LGE) imaging was performed at least 10 minutes following the contrast agent ‘top-up’.

### CMR analysis

LV mass, end-diastolic volume, end-systolic volume and ejection fraction (EF) were quantified from steady-state free precession images using CMRtools (Cardiovascular Imaging Solutions, UK) [7]. Peak systolic circumferential strain (εcc) and strain rate (systolic and early-diastolic) were measured from mid-ventricular short-axis tagged images using SinMod (inTag, CREATIS lab, France, and Maastricht University, The Netherlands, v5.0) [8]. Basal and apical short-axis rotation, calculated from tagged images using the same software, and epicardial areas, were incorporated into a custom-written algorithm (Microsoft Excel using Visual Basic) in order to calculate twist (basal rotation–apical rotation), normalized twist (twist angle divided by distance between slices) and torsion (circumferential-longitudinal shear angle; calculated by multiplying normalized twist by mean of basal and apical epicardial radii).

Myocardial T_1_ relaxation time was measured as described previously [9]. Briefly, voxel-wise T_1_ relaxation maps (‘T1 mapping’) were obtained from the MOLLI images using a 3 parameter fit to signal intensity, S as a function of effective inversion time (TIeff) according to S(TIeff) = A - Be^(−TIeff/T1*)^ and T_1_ was calculated as T_1_ = T_1_*((B/A)-1). After applying a heart-rate correction algorithm, mean mid-ventricular pixel T_1_ relaxation times before and after contrast were then used to calculate myocardial extracellular volume (ECV) according the following formula: ECV = λ × (1- haematocrit). Where the partition coefficient, λ = ΔR_1_(myocardium)/ΔR_1_(blood). ΔR_1_ is proportional to contrast agent concentration. ΔR_1_ = R_1_(post-contrast) – R_1_(pre-contrast).

Voxel-wise T_2_ relaxation maps (‘T2 mapping’) were obtained from the T2-prepared SSFP images using a linear least squares fit to the log transformed signal intensity in each voxel according to In(S) = In(S_0_) – (T2_prep_/T_2_) where S is the measured signal intensity and S_0_ is the fitted signal intensity corresponding to no T_2_-preparation (*T2*_*prep*_ = 0).

Perfusion quantification was performed as described previously [6]. Briefly, signal intensity curves were extracted from the average signal in the blood pool, to provide an arterial input function, and on a voxel-wise basis from myocardial regions of interest. Signal intensity was converted to contrast agent concentration [10]. Perfusion values were obtained on a voxel-wise basis using generalized Tikhonov deconvolution with a b-spline representation of the impulse response function [11]. Myocardial perfusion reserve (MPR) was calculated by dividing median hyperaemic myocardial blood flow (MBF) by median resting MBF. LGE images were reported visually by 2 experienced operators and the presence or absence of LGE, and its distribution pattern, were recorded.

### Endomyocardial biopsy and histological analysis

Right ventricular biopsy was performed in a standard manner. Five to ten tissue samples were obtained from the right ventricular septum, stained with haematoxylin and eosin and analysed with light microscopy. Tissue was graded according to the 2005 Revision of the International Society for Heart and Lung Transplantation (ISHLT) Standardized Cardiac Biopsy Grading Criteria [12]. Immunosuppressive therapy is generally augmented at grade 2R or higher and thus grades 2R – 3R were considered ‘significant’ ACAR and grades 0R – 1R ‘non-significant’ ACAR. As is the clinical policy at our Institution, immunopathological assessment was performed if histological features of antibody-mediated rejection were present or if there was a high clinical suspicion of ACAR in the absence of significant acute cellular rejection. Patients treated for ACAR on the basis of high clinical suspicion alone (i.e. in the absence of ≥ grade 2R acute cellular rejection or antibody mediated rejection) were also recorded. Patients were followed up in order to determine ACAR grade on the biopsy subsequent to those included in study.

### Statistical analysis

All data was analysed in a blinded fashion, with independent analysis of CMR and biopsy data. Statistical analysis was performed using SPSS (IBM, USA; v20). Continuous variables are expressed as mean ± SD unless stated. An independent-samples t test (or Mann–Whitney U test where appropriate) was used to compare baseline transplant patient and healthy volunteer demographic data. CMR data were assessed according to ACAR grade using generalized estimating equations (GEE) with an ordinal logistic model in order to adjust for the repeated measurements over time within in each subject. Subsequently, CMR data were dichotomized according to the presence (≥ grade 2R) or absence (grade 0R – 1R) of significant ACAR and compared using GEE with a binary logistic model. CMR data from healthy volunteers and transplant patients without significant ACAR were compared using the same method. A GEE linear regression model was used to compare CMR data between scans performed at different time points, and also to assess the relationship between CMR parameters. CMR data from transplant patients at a single time-point were compared with data from healthy volunteers using an independent t test.

## Results

### Study population and biopsy results

Twenty-two patients were recruited (Figure 1). Demographic data are presented in Table 1. Each patient underwent CMR on the same day as biopsy on four separate occasions i.e. 88 biopsies and corresponding CMR scans in total. Median (IQR) timing of the four scans was 6.9 (4.9-8.3) weeks, 10.9 (9.5-12.6) weeks, 16.6 (13.2-19.6) weeks and 22.3 (19.6-26.3) weeks post-transplantation. Five patients (23%) displayed grade 2R ACAR on biopsies included in the study. Three of these patients demonstrated grade 2R ACAR on 2 separate occasions. Of the 88 biopsies included in the study, 48 (55%) were grade 0R, 29 (33%) were grade 1R, 8 (9%) were grade 2R, 0 were grade 3R and 3 (3%) were inadequate (insufficient tissue to allow accurate interpretation). No biopsy demonstrated antibody mediated rejection and no patient was treated for ACAR on the basis of high clinical suspicion alone. Eight (36%) patients underwent CMR without contrast agent (5 patients had severe renal dysfunction and 3 patients refused consent). Healthy volunteers and transplant recipients were well matched (Table 1).

**Table 1:** Baseline subject characteristics

	**Transplant patients (n = 22)**	**Healthy volunteers (n = 10)**	**p value**
Male	17 (77%)	7 (70%)	0.660
Age (years)	49 ± 10	49 ± 8	0.961
Non-white	2 (9%)	1 (10%)	0.676
BSA (m^2^)	1.86 ± 0.20	1.94 ± 0.19	0.290
BMI (kg/m^2^)	24.7 ± 3.6	25.7 ± 4.1	0.484
eGFR (mL/min/m^2^)	61 ± 24	89 ± 13	<0.001
HR (bpm)	89 ± 15	60 ± 6	<0.001
Systolic BP (mmHg)	135 ± 17	111 ± 8	<0.001
Indication for transplant			
DCM	12 (55%)	-	
IHD	6 (27%)	-	
ARVC	2 (9%)	-	
HCM	1 (5%)	-	
Re-transplant	1 (5%)	-	
Pregnancy prior to transplantation	3 (14%)	-	
VAD prior to transplantation	1 (5%)	-	
Donor age (years)	42 ± 7	-	
Donor male	17 (77%)	-	
Donor: recipient gender match	18 (82%)	-	
Donor cause of death			
ICH	13 (59%)	-	
Head trauma	5 (23%)	-	
Other	4 (18%)	-	
CMV donor pos., recipient neg.	5 (23%)	-	
Ischaemic time (min)	184 ± 52	-	
Induction immunosuppression			
rATG	21 (95%)	-	
Basiliximab	1 (5%)	-	
Initial immunosuppression regime			
Cyclosporine/prednisolone/MMF	21 (95%)	-	
Tacrolimus/prednisolone/MMF	1 (5%)	-	
ICU stay (days)	17 ± 19	-	
Hospital stay (days)	36 ± 23	-	

BSA indicates body surface area; BMI body mass index; eGFR estimated glomerular filtration rate; HR heart rate; BP blood pressure; DCM indicates dilated cardiomyopathy; IHD ischemic heart disease; ARVC arrhythmogenic right ventricular cardiomyopathy; HCM hypertrophic cardiomyopathy; (Re-transplant: this patient suffered immediate graft failure and therefore underwent a second transplantation); VAD ventricular assist device; ICH spontaneous intracranial haemorrhage; CMV cytomegalovirus; rATG rabbit antithymocyte globulin; MMF Mycophenolate mofetil; ICU intensive care unit.

### ACAR

**Figure 2:**
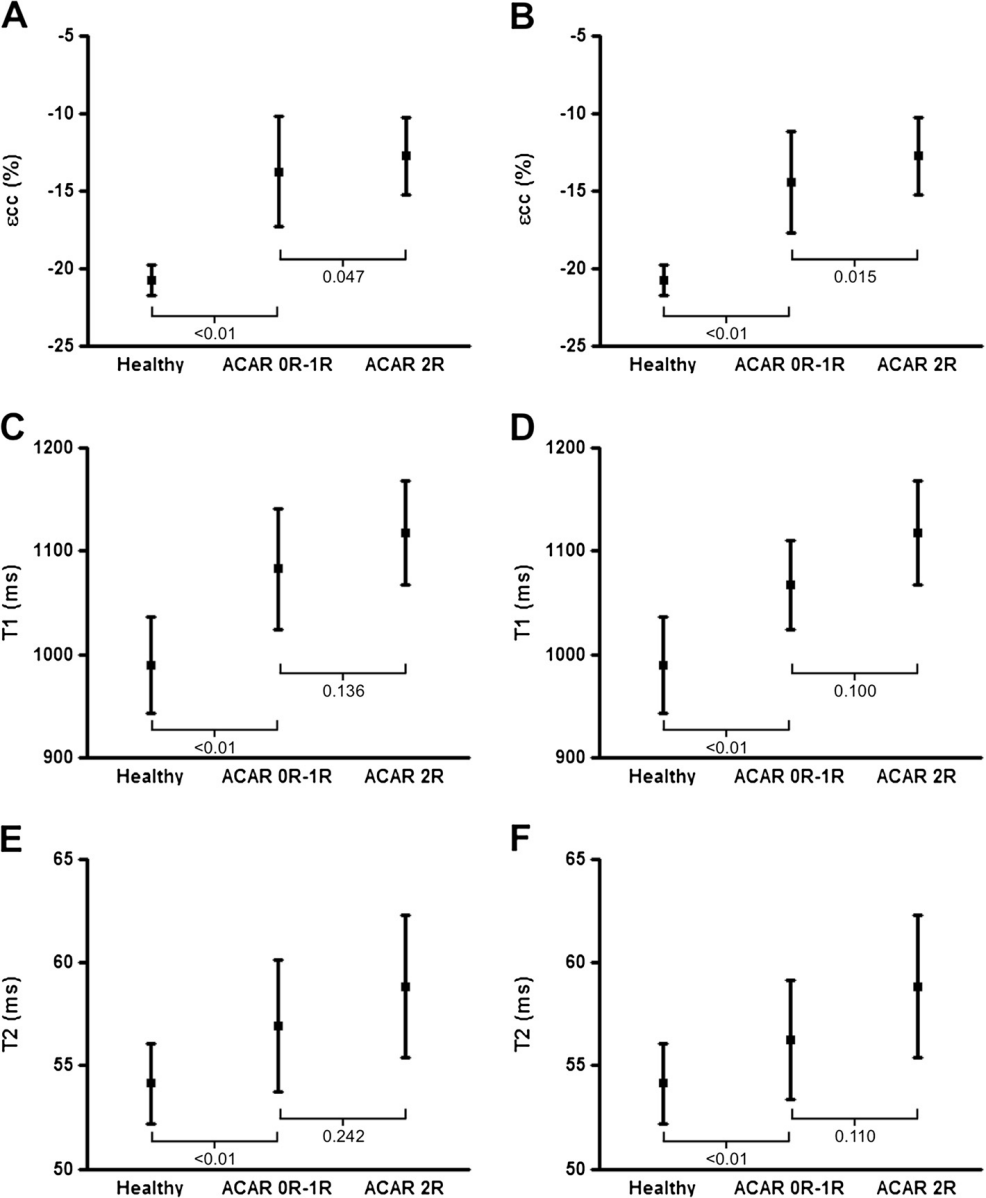
**CMR parameters in significant and non-significant rejection and healthy volunteers.** Peak systolic circumferential strain (εcc, **A**), myocardial T_1_ relaxation time **(C)** and myocardial T_2_ relaxation time **(E)** in significant (grade 2R) and non-significant (grades 0R-1R) acute cardiac allograft rejection (ACAR) and in matched healthy volunteers. **B**, **D** and **F** display corresponding data after patients with primary graft dysfunction have been excluded.

**Figure 3:**
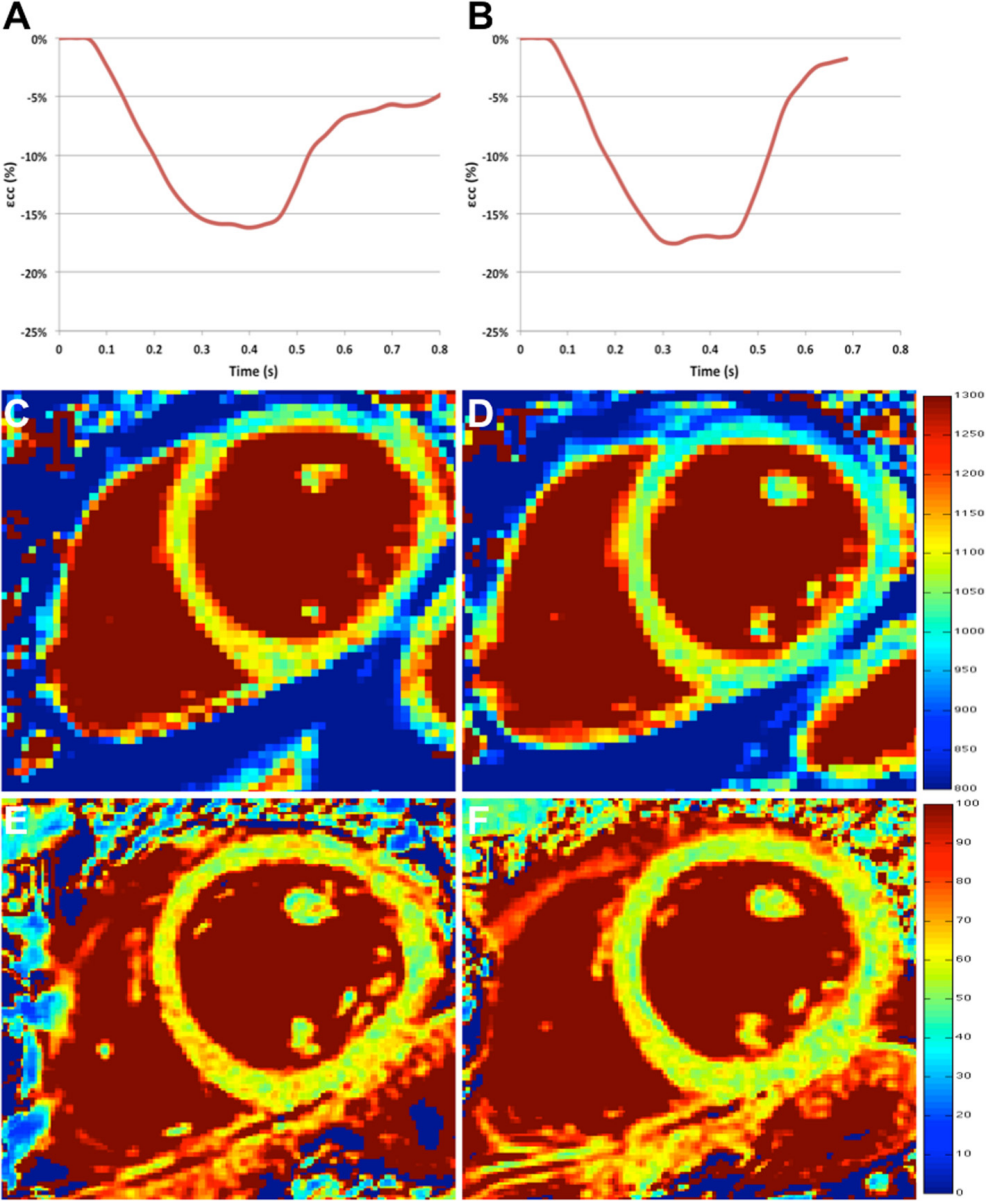
**Example CMR findings.** Example circumferential strain graphs **(AandB)**, T_1_ maps **(CandD)** and T_2_ maps **(EandF)** in in a patient with grade 2R **(A, C, E)** and the corresponding findings in the same patient on the subsequent CMR scan performed 5 weeks later after treatment when ACAR grade was 0 **(B, D, F)**.

Multiparametric CMR data is presented in Table 2. Whilst εcc, native T_1_ and T_2_ data demonstrated a trend towards a deterioration as ACAR severity increased, only εcc showed a significant difference between significant and non-significant ACAR, although the absolute difference was small and there was considerable overlap between groups (Table 2, Figures 2 and 3). The outcome was no different if analysis was performed using CMR data from septal segments only. Pericardial effusion, seen on 9 scans (10%), was unrelated to ACAR (p = 0.864). Only one biopsy demonstrating significant ACAR was accompanied by a CMR scan that was performed with contrast agent, hence meaningful assessment of perfusion, LGE and ECV data with regards to ACAR was not possible.

**Table 2:** Cardiovascular magnetic resonance findings according to the presence (grade 2R) or absence (grade 0R-1R) of significant acute cardiac allograft rejection

	**Healthy volunteers**	**No rejection (0R – 1R)**	**Rejection (2R)**	**p value**
LVEDVI (mL/m^2^)	85.9 ± 8.1	84.2 ± 21.7	73.6 ± 15.7	0.287
LVESVI (mL/m^2^)	28.5 ± 4.3	35.5 ± 21.9	28.1 ± 10.9	0.329
LVEF (%)	66.9 ± 4.2*	60.4 ± 13.7	62.2 ± 9.3	0.655
LVMI (g/m^2^)	46.4 ± 7.3^†^	62.2 ± 58.4	58.4 ± 7.3	0.362
εcc (%)	−20.7 ± 1.0^†^	−13.7 ± 3.6	−12.7 ± 2.5	0.047
Peak systolic SR (1/s)	−1.17 ± 0.14	−1.05 ± 0.31	−1.06 ± 0.26	0.568
Early diastolic SR (1/s)	0.37 ± 0.20*	0.25 ± 0.13	0.26 ± 0.13	0.917
Normalised twist (°/mm)	0.34 ± 0.12*	0.26 ± 0.10	0.29 ± 0.08	0.151
Torsion (mm)	8.99 ± 3.11	7.49 ± 2.86	8.11 ± 1.93	0.237
Time to peak torsion (%AVC)	96.4 ± 5.5	97.7 ± 8.6	100.6 ± 8.0	0.555
Native T_1_ (ms)	989 ± 46^†^	1083 ± 59	1118 ± 51	0.136
T_2_ (ms)	54.1 ± 2.0^†^	57.0 ± 3.2	58.8 ± 3.5	0.242

p values refer to the significance of the difference between no rejection and rejection. The significance of the difference between healthy volunteers and no rejection is denoted by *(p < 0.05) or ^†^(p < 0.01). LV indicates left ventricle; EDV end diastolic volume; ESV end-systolic volume; SV stroke volume; EF ejection fraction, εcc peak systolic circumferential strain; SR strain rate; %AVC refers to time between peak electrocardiogram R wave and aortic valve closure expressed as a percentage. The suffix I indicates indexed to body surface area.

Six of the 8 subsequent biopsies to those showing significant ACAR demonstrated non-significant ACAR after immunosuppressive treatment and had corresponding CMR data (one patient demonstrated grade 2R on 2 consecutive biopsies and one patient showed grade 2R ACAR on biopsy number 4 hence no follow-up CMR data was available). On these ‘convalescent’ scans, CMR parameters were seen to return to ‘baseline’ levels (εcc: −15.0 ± 2.5%, native T_1_: 1048 ± 36 ms, T_2_: 55.6 ± 3.4 ms).

Five biopsies demonstrating non-significant ACAR were followed by a subsequent biopsy performed within 28 days that demonstrated grade 2R. CMR data corresponding to these biopsies demonstrated εcc (−13.5 ± 2.2%), native T_1_ (1090 ± 56 ms) and T_2_ (58.8 ± 3.1 ms) values that were intermediate to values for non-significant and significant ACAR.

### Time from transplantation

**Figure 4:**
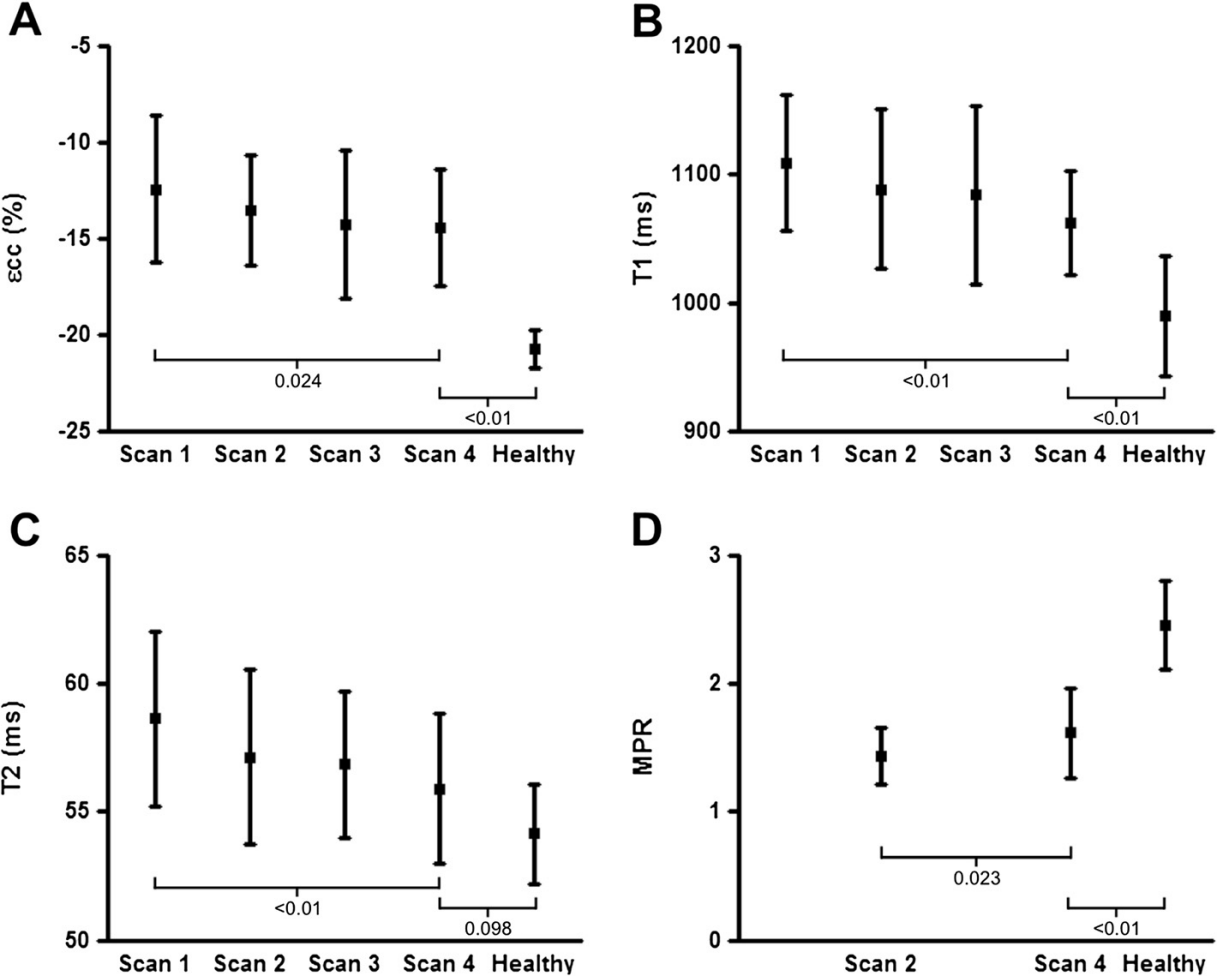
**Change in CMR parameters over time from transplantation.** Change in peak systolic circumferential strain (εcc, **A**), myocardial T_1_ relaxation time **(B)**, myocardial T_2_ relaxation time **(C)** and myocardial perfusion reserve (MPR, D) over time from transplantation (scan 1: 6.9 weeks post-transplantation; scan 2: 10.9 weeks; scan 3: 16.6 weeks; scan 4: 22.3 weeks) and comparison of parameters at the time of scan 4 with matched healthy volunteers.

CMR findings according to scan number (i.e. time from transplantation) are displayed in Table 3. LV mass, εcc, native T_1_ and T_2_ decreased significantly, and MPR increased significantly, between scans 1 and 4 (Figure 4). Seven patients (32%) demonstrated a pericardial effusion on scan 1, which had resolved in all but 2 patients by scan 2; pericardial effusion was not observed on scans 3 and 4 in any patient (p = 0.002). On LGE imaging, one patient displayed evidence of an anterior myocardial infarction and another patient demonstrated right ventricular insertion point enhancement, but no temporal changes were observed. At the time of scan 4, LV volumetrics, EF, SR and T_2_ were not significantly different between transplant recipients and healthy volunteers, although other parameters remained abnormal.

**Table 3:** CMR findings displayed according to scan number

	**Scan 1 (6.9 weeks)**	**Scan 2 (10.9 weeks)**	**Scan 3 (16.6 weeks)**	**Scan 4 (22.3 weeks)**	**p value**	**Healthy volunteers**
LVEDVI (mL/m^2^)	83.2 ± 20.3	83.4 ± 23.2	83.7 ± 22.6	82.6 ± 19.9	0.979	85.9 ± 8.1
LVESVI (mL/m^2^)	35.3 ± 21.1	35.8 ± 23.6	34.5 ± 21.2	33.5 ± 19.4	0.423	28.5 ± 4.3
LVEF (%)	59.5 ± 13.2	60.1 ± 14.2	61.1 ± 13.9	61.3 ± 12.2	0.216	66.9 ± 4.2
LVMI (g/m^2^)	63.6 ± 15.7	62.5 ± 12.9	62.1 ± 12.8	59.3 ± 11.9	0.001	46.4 ± 7.3^†^
εcc (%)	−12.4 ± 3.8	−13.5 ± 2.9	−14.2 ± 3.8	−14.4 ± 3.0	0.024	−20.7 ± 1.0^†^
Peak systolic SR (1/s)	−1.05 ± 0.33	−1.03 ± 0.27	−1.07 ± 0.33	−1.05 ± 0.30	0.669	−1.17 ± 0.14
Early diastolic SR (1/s)	0.29 ± 0.13	0.25 ± 0.10	0.23 ± 0.12	0.25 ± 0.15	0.499	0.37 ± 0.20
Normalised twist (°/mm)	0.26 ± 0.10	0.25 ± 0.10	0.27 ± 0.11	0.27 ± 0.08	0.709	0.34 ± 0.12*
Torsion (mm)	7.68 ± 2.89	7.16 ± 2.73	7.78 ± 3.19	7.59 ± 2.24	0.761	8.99 ± 3.11
Time to peak torsion (% AVC)	101.0 ± 11.0	97.1 ± 7.2	97.9 ± 6.3	96.5 ± 9.3	0.466	96.4 ± 5.5
Native T_1_ (ms)	1109 ± 53	1089 ± 62	1084 ± 70	1063 ± 40	<0.001	989 ± 46^†^
T_2_ (ms)	58.7 ± 3.4	57.1 ± 3.4	56.9 ± 2.9	55.9 ± 2.9	0.003	54.1 ± 2.0
Resting MBF (mL/min/g)	-	0.85 ± 0.12	-	0.86 ± 0.16	0.150	0.74 ± 0.10
Stress MBF (mL/min/g)	-	1.21 ± 0.18	-	1.37 ± 0.26	0.172	1.81 ± 0.29^†^
MPR	-	1.44 ± 0.22	-	1.62 ± 0.35	0.023	2.46 ± 0.34^†^
ECV	-	30.1 ± 0.5	-	28.0 ± 1.6	<0.001	25.3 ± 0.18^†^

Time between transplantation and scan is given in brackets.

p values refer to the significance of the difference between scans. The significance of the difference between healthy volunteers and scan 4 is denoted by *(p < 0.05) and ^†^(p < 0.01). MBF indicates myocardial blood flow; MPR myocardial perfusion reserve; LGE late gadolinium enhancement; ECV myocardial extracellular volume. Other abbreviations as per Table 2.

### Influence of other factors on CMR findings

Three patients (14%) developed primary graft dysfunction (PGD). In these patients, mean baseline EF was 26.2 ± 4.9% (versus 63.0 ± 7.5% in patients without graft dysfunction, p < 0.001) and remained severely impaired across all 4 scans, albeit with some improvement (Table 4). Measures of contractile function and tissue characterization parameters were also markedly abnormal in these patients and were significantly different to patients without PGD (Table 4). In light of these findings, analysis of CMR data with regards to ACAR was repeated after adjusting for PGD, with results as follows (non-significant ACAR vs. significant ACAR): εcc 14.4 ± 3.2% vs. 12.7 ± 2.5%, p = 0.015; native T_1_ 1067 ± 43 ms vs. 1118 ± 51 ms, p = 0.100; T_2_ 56.3 ± 2.7 ms vs. 58.8 ± 3.5 ms, p = 0.110 (Figure 2).

**Table 4:** Selected CMR parameters displayed according to the presence of primary graft dysfunction (PGD)

	**Scan 1 (6.9 weeks)**	**Scan 2 (10.9 weeks)**	**Scan 3 (16.6 weeks)**	**Scan 4 (22.3 weeks)**	**p value**
LVEDVI (mL/m^2^)					
No PGD	78.5 ± 14.6	76.4 ± 15.5	77.4 ± 16.5	77.7 ± 14.1	
PGD	127.5 ± 16.6^†^	127.3 ± 12.1^†^	123.7 ± 10.4^†^	123.6 ± 12.8^†^	<0.001
LVESVI (mL/m^2^)					
No PGD	29.1 ± 8.3	27.3 ± 8.9	27.0 ± 9.5	27.9 ± 9.2	
PGD	94.2 ± 7.5^†^	89.4 ± 11.1^†^	81.7 ± 5.8^†^	81.4 ± 17.9^†^	<0.001
LVEF (%)					
No PGD	63.0 ± 7.5	64.8 ± 7.7	65.4 ± 8.7	64.5 ± 7.9	
PGD	26.2 ± 4.9^†^	29.9 ± 2.2^†^	33.7 ± 5.8^†^	34.5 ± 7.8^†^	<0.001
εcc (%)					
No PGD	−13.1 ± 3.6	−14.0 ± 2.6	−15.0 ± 3.5	−14.9 ± 2.9	
PGD	−8.5 ± 3.3*	−9.3 ± 1.7*	−9.3 ± 1.6*	−10.9 ± 0.6	<0.001
Native T_1_ (ms)					
No PGD	1099 ± 42	1067 ± 42	1066 ± 59	1055 ± 32	
PGD	1167 ± 79*	1184 ± 66^†^	1177 ± 50^†^	1122 ± 66*	0.001
T_2_ (ms)					
No PGD	58.0 ± 2.88	56.6 ± 3.2	56.2 ± 2.7	55.3 ± 2.3	
PGD	64.7 ± 0.93^†^	60.5 ± 3.4	60.3 ± 0.5*	60.8 ± 3.0^†^	<0.001

p values refer to the significance of the difference in each CMR parameter between the presence and absence of PGD taking into account all time points, assessed using generalized estimating equations. The significance of the difference in each CMR parameter between the presence and absence of PGD at each individual time point, assessed using independent t tests, is denoted by *(p < 0.05) or ^†^(p < 0.01). Abbreviations as per Table 2.

Eight patients (36%) were treated for CMV infection while taking part in the study (20 biopsies performed during CMV treatment). After adjusting for PGD, no CMR parameter differed significantly according to CMV infection.

### Relationship between CMR parameters

There were significant linear relationships between LVEF and εcc (LVEF = −0.49εcc + 53; p = 0.044), LVEF and peak systolic SR (LVEF = −8.9SR + 51; p = 0.016), LVEF and T_1_ (LVEF = −0.04T_1_ + 106; p = 0.006), LVEF and T_2_ (LVEF = −0.65T_1_ + 97; p = 0.005), T_1_ and T_2_ (T_1_ = 7.9T_2_ + 629; p < 0.001); T_1_ and εcc (T_1_ = 4.5εcc + 1148; p = 0.004); T_2_ and εcc (T_2_ = 0.43εcc + 63; p < 0.001) and T_2_ and peak systolic SR (T_2_ = 4.2SR + 61; p = 0.002).

## Discussion

In this study CMR was not able to accurately detect ACAR in the early phase post-transplantation. However, this study does demonstrate the complexity of factors affecting allograft structure and function in this period and provides novel insight into the myocardial injury associated with transplantation, and its recovery.

Most published studies investigating the role of non-invasive ACAR surveillance techniques select patients known/suspected to have ACAR, and commonly include patients outside the time period when the early detection of ACAR is likely to be most useful [13]. In contrast, the current study used an unselected cohort and focused on the time period when ACAR is of greatest clinical importance (i.e. the first 6 post-operative months) [1].

The per-patient incidence of significant ACAR in the current study (23%) is in keeping with that reported in the most recent data from the ISHLT Registry [1]. Nevertheless, in keeping with other contemporary studies in this field, the number of episodes of significant ACAR captured (9%) is relatively small and reflects the decreasing incidence of ACAR secondary to advances in immunosuppression [14]. Indeed, while the decreasing incidence of ACAR makes biopsy increasingly unattractive (the yield of biopsy is now of the same order of magnitude as its complication rate), the decreasing incidence makes the assessment of new surveillance techniques more difficult [2].

In the present study εcc was significantly lower in grade 2R ACAR compared to grades 0R and 1R, although the absolute difference was small and there was considerable overlap between groups, indeed by considering all data points as independent (i.e. by not taking into account the repeated measurements within each patient) which allows receiver operating characteristic (ROC) curve analysis to be performed, sensitivity and specificity of εcc for detecting significant ACAR were only 75% and 64% respectively (area under curve (AUC) 0.69, 95% confidence intervals 0.53-0.85). In a rodent transplant model, Wu et al. [15] found regional impairment of εcc, as assessed with CMR tagging, to correspond to areas of macrophage infiltration, however echocardiographic data in humans regarding the utility of strain (in all orthogonal directions) for detecting ACAR is inconsistent [16],[17].

Myocardial T_1_ and T_2_ relaxation times are sensitive to changes in myocardial water content and have been proposed to detect myocardial oedema in other conditions [18]-[20]. However, in the current study, myocardial T_1_ and T_2_ were not significantly higher in grade 2R ACAR compared to grades 0R-1R, although both demonstrated trends towards higher values, particularly after accounting for PGD.

In a recent study by Usman et al. [14], ACAR was associated with elevated myocardial T_2_, which is in keeping with the findings of an early CMR study by Marie et al. [21]. However, there are important differences between these studies and the current study. In the studies by Usman et al. and Marie et al. patients were substantially longer post-transplant than in the current study, thus reducing the effect of transplant-related myocardial injury, described below, on T_2_ measurements, but also missing the window in which early detection of ACAR is thought to be most useful (also see below). In addition, both studies specifically selected patients known/suspected of having ACAR and different definitions of ‘significant’ ACAR were used. Finally, neither study made statistical adjustment for repeated measurements within the same patients (for example 33 patients in the study by Marie et al. underwent 2 – 4 CMR scans). Indeed if equivalent statistical methods to those used by Usman and Marie are applied here, the differences in T_2_ (and T_1_) between ACAR groups after accounting for PGD become significant (T_2_: p = 0.016; T_1_: p = 0.008) although the sensitivity and specificity remain modest (T_2_: 75% and 67% respectively, AUC 0.74 (0.53-0.95); T_1_: 83% and 72% respectively, AUC 0.79 (0.59-0.98)). The role of myocardial T_1_ in ACAR has not been previously evaluated using contemporary CMR techniques.

Whilst recognizing that CMR methods requiring gadolinium contrast agent would be undesirable for ACAR surveillance, post-contrast CMR techniques were included in order to provide further characterization of ACAR pathophysiology. However the size of the cohort studied and prevalence of renal impairment, typical of many studies involving transplant patients, meant insufficient patients with ACAR underwent contrast-enhanced CMR to allow meaningful comparison of these parameters.

This study does serve to provide detailed characterization of the evolution of LV structure and function during the early phase post-transplantation. Over the first 5 post-operative months significant improvements were seen in markers of LV structure (mass) and contractility (εcc), proposed markers of myocardial oedema (native T_1_, T_2_ and ECV) and microvascular function (MPR), although few parameters normalized.

The insults to which the donor heart is subjected in the peri-transplant period, including brain death and its sequelae, ischemia and reperfusion, are likely to cause considerable myocardial injury, despite the preservation of gross markers of cardiac function (e.g. EF) in most patients. The current study comprehensively characterizes this myocardial injury for the first time, suggesting that it manifests as myocardial oedema, microvascular dysfunction and, likely as a consequence of both of these factors and of direct myocyte injury, impaired contractile function. The study also provides insight into its natural history, demonstrating how the injury improves over the first 5-months post-transplant but also showing that it persists for at least this period, with few parameters returning to normal over this time. Furthermore it is of considerable interest to note that proposed markers of oedema were significantly higher in patients that developed PGD compared to other allograft recipients, and remained elevated over the period studied. The mechanisms of myocardial stunning, seen in ischemic heart disease, include myocardial oedema; possibly via increasing the distance between actin and myosin filaments, which in turn leads to reduced contractility [22],[23]. The pathophysiological mechanisms of PGD are not well understood and are likely multifactorial, but the results of this study suggest that, analogous to myocardial stunning, oedema may play an important role.

There has been little previous investigation into temporal changes in allograft structure and function in the early phase post-transplant. Using echocardiography, Antunes et al. [24] found LV EF to improve significantly over the first month post-transplantation. Eleid et al. [17] found εcc, assessed using serial speckle-tracking echocardiography, remained markedly impaired compared to healthy subjects throughout the first two post-operative years although degree of temporal change was not reported. Wisenburg et al. [25] using spin echo sequences at 0.15T, found myocardial T_1_ and T_2_ to be elevated in all patients during the very early period post-transplant however values returned to normal 25 days post-transplant. In the current study myocardial T_1_ and T_2_ remained elevated for considerably longer, which is in keeping with contemporary CMR findings in other pathologies such as myocardial infarction or myocarditis [23]. Finally in keeping with the current study, Preumont et al. [26] demonstrated higher MPR, as assessed using Nitrogen-13 PET, in patients with angiographically normal epicardial coronary arteries scanned at 9-months post-transplant compared to matched patients scanned at 3-months post-transplant.

Taking the findings of the current study and of Usman et al. [14] together it may be that CMR parameters become more useful for detecting ACAR as time from transplantation increases and the transplant-related myocardial injury subsides. The paradox however is that while non-invasive approaches to ACAR surveillance may become more discriminatory as time from transplantation increases, the benefit of the early detection of ACAR diminishes, indeed the usefulness of routine screening later than one year post-transplant is subject to debate [2],[27].

### Limitations

Despite over 2 years of recruitment and a recruitment rate of over 75% in those eligible, the number of patients included is relatively small. This is in part reflective of the robust study design, although the size of the cohort and number of scans performed here are in keeping with many studies assessing non-invasive approaches to ACAR surveillance. As acknowledged earlier, the number of episodes of significant ACAR captured is also relatively small. It is also recognized that biopsy is limited as a reference standard, with ‘biopsy-negative’ ACAR widely reported [3], however patients were followed-up in order to identify those treated for ACAR in the absence of positive biopsy. Baseline post-transplant coronary angiography was not performed and as such epicardial coronary disease cannot be excluded as a cause of the low MPR seen in transplant recipients, however given that MPR improved significantly over time this is unlikely. Finally, as is well documented elsewhere, histological validation of T1 and T2 imaging for detecting and quantifying non-infarct related myocardial oedema is lacking. Nevertheless, our application of T1 and T2 sequences is in keeping with contemporary literature.

## Conclusions

In this study, multiparametric CMR demonstrated that multiple factors affect cardiac allograft structure and function in the early phase post-transplantation. Whilst CMR provided novel insight into the myocardial injury associated with transplantation, it was not able to accurately detect ACAR as diagnosed by biopsy during this period.

## Abbreviations

ACAR: Acute cardiac allograft rejection

CMR: Cardiovascular magnetic resonance

ECV: Myocardial extracellular volume

EF: Ejection fraction

LGE: Late gadolinium enhancement

LV: Left ventricle

MBF: Myocardial blood flow

MPR: Myocardial perfusion reserve

PGD: Primary graft dysfunction

ROC: Receiver operating characteristic

## Competing interests

The authors declare that they have no competing interest.

## Authors’ contributions

All authors have contributed significantly to the work. CAM and MS conceived and directed the project. JHN and CAM developed the CMR analysis tools with input from GC. CAM, SMS, NY and SGW recruited the patients. DC, CAM, MPA and MS performed the CMR scanning and analysis. PB performed the histological analysis. All authors provided critical review of the manuscript. All authors read and approved the final manuscript.
